# Case Report: Anti-LGI1 Limbic Encephalitis Associated With Anti-thyroid Autoantibodies

**DOI:** 10.3389/fneur.2020.620483

**Published:** 2021-01-15

**Authors:** Ricardo Otiniano-Sifuentes, Anali Cuba Antezana, Walter F. De La Cruz Ramirez, Kevin Pacheco-Barrios, Darwin A. Segura Chavez

**Affiliations:** ^1^Department of Neurovascular Diseases, Instituto Nacional de Ciencias Neurológicas, Lima, Peru; ^2^Servicio de Neurología y Neuropsiquiatría, Departamento de Medicina, Hospital Nacional Cayetano Heredia, Lima, Peru; ^3^Departamento de Investigación, Docencia y Atención Especializada en Epilepsia, Instituto Nacional de Ciencias Neurológicas, Lima, Peru; ^4^Unidad de Investigación para la Generación y Síntesis de Evidencias en Salud, Universidad San Ignacio de Loyola, Lima, Peru; ^5^Centro Básico de Investigación en Enfermedades Neuromusculares, Instituto Nacional de Ciencias Neurológicas, Lima, Peru

**Keywords:** autoimmune encephalitis, limbic encephalitis, anti-LGI1, anti-thyroid autoantibodies, hashimoto's encephalopathy

## Abstract

Anti-LGI1 encephalitis is an autoimmune encephalitis with antibodies against leucine-rich glioma-inactivated 1 (LGI1), first described in 2010. It is a non-frequent and poorly understood entity that represents the second most frequent cause of autoimmune encephalitis. This entity is characterized by the presence of limbic encephalitis, hyponatremia, and faciobrachial dystonic seizures. Herein, we present the case of a male patient with an onset of epileptic seizures (generalized tonic-clonic seizure), and involuntary dystonic movements that affect the right side of his face and right upper limb associated with mental disorder, and affectation of higher functions. The electroencephalogram showed continuous generalized slowing of the background activity. The brain magnetic resonance imaging showed signal hyperintensity at the level of both mesial temporal lobes and hippocampi and in the head of the right caudate nucleus. Anti-thyroglobulin antibodies were positive, and he was initially diagnosed as Hashimoto's encephalopathy (HE). However, the response to corticosteroids was not completed as it is usually observed in HE. For that, antibodies for autoimmune encephalitis were tested, and the anti-LGI1 antibodies were positive in serum and cerebrospinal fluid. HE is an important differential diagnosis to consider. Furthermore, the presence of Anti-thyroglobulin antibodies should not be taken as the definitive diagnostic criteria, since these antibodies could be associated with other autoimmune encephalopathies, which include in addition to anti-LGI1, anti-NMDA and anti-Caspr2.

## Background

Anti-LGI1 encephalitis is an autoimmune encephalitis with antibodies against leucine-rich glioma-inactivated 1 (LGI1), first described in 2010 (1). It is a subacute disorder characterized by presenting symptoms of limbic encephalitis such as memory deterioration, epileptic seizures, and behavioral disturbances associated with faciobrachial dystonic seizures (FBDS) and hyponatremia (2, 3). LGI1 limbic encephalitis occurs between the ages of 30–80, being more frequent in males. Changes in brain magnetic resonance imaging (MRI) are usually found in up to 84%. The most frequently affected areas are the medial temporal lobe and the basal ganglia (BG). The cerebrospinal fluid (CSF) study is often normal. However, there may be slight inflammatory changes or the presence of oligoclonal bands (4).

Due to the presence of neuropsychiatric symptoms, Hashimoto's encephalopathy (HE) can be considered as a differential diagnosis. Therefore, the test for antithyroid antibodies (anti-Ty Ab) is necessary. However, the coexistence of these antibodies in other autoimmune diseases, including limbic encephalitides such as anti-NMDAr, Caspr2, and LGI1, must be consider as a potential source of misdiagnosis (5). We present the case of a patient with anti-LG1 encephalitis with the coexistence of anti-Ty Ab initially classified as HE.

## Case Presentation

A 77-year-old male with a history of controlled hypertension was brought to our office with an 11-months history that began with a 5-min generalized tonic-clonic seizure (GTCS). At that time, he was therefore treated with phenytoin 100 mg every 8 h. After that he remained asymptomatic. Two months later he presented mental disorder such as irritability and apathy at times. Four months after the symptoms began, altered mental status were added, as well as episodic memory deficit and sleep inversion. These symptoms progress, and after 6 months of illness, he also presented visual hallucinations and involuntary dystonic movements that affect the right side of his face and right upper limb. The episodes occurred several times during the day only seconds long. Two months before admission, apathy progressed, reaching a state of akinetic mutism, and again presented GTCS, for which he is brought to the emergency department.

On neurological examination during hospitalization, the patient had spontaneous ocular opening with poor eye contact, unable to follow simple commands, slurred speech with a tendency to mutism, there was no motor deficit, but he had mild limb mobility, and indifferent plantar reflexes bilaterally. At times he had oromandibular movements associated with myoclonus of the right upper limb.

**Figure 1:**
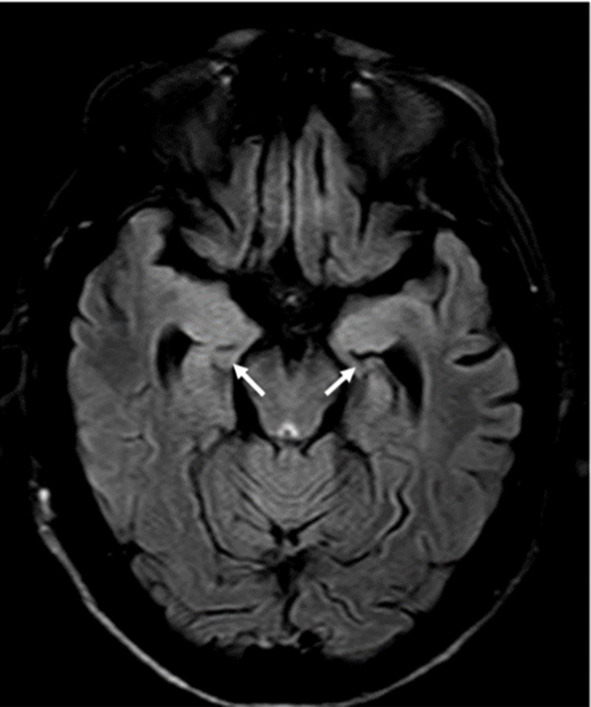
Brain MRI FLAIR protocol, showing signal hyperintensity, and atrophy at the mesial temporal level and bilateral hippocampi.

**Figure 2:**
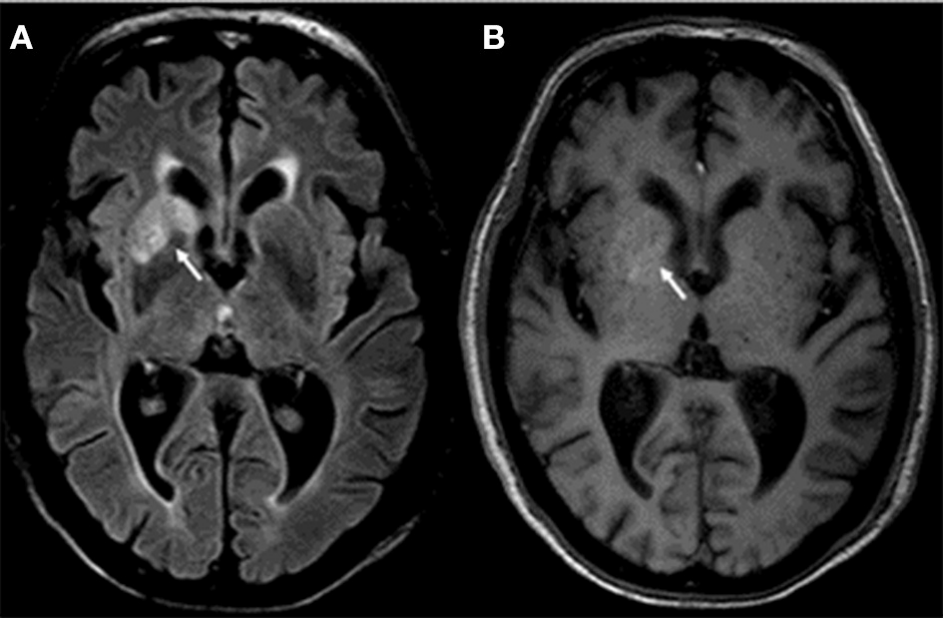
Brain MRI shows signal hyperintensity at the level of the basal nuclei (caudate nucleus head and putamen) on the right side in the FLAIR protocol **(A)**. Discrete hyperintensity is also observed in the same region in the T1 protocol **(B)**.

He had no abnormalities in the hematological exams. Liver profile, lipids, vitamin B12, HIV, and RPR tests were performed without any abnormal finding. Hyponatremia was found (with values between 125 and 135 mEq/L). The electroencephalogram (EEG) showed continuous generalized slowing of the background activity. CSF study revealed normal proteins (17 mg/dL; reference range: 15–45 mg/dL), glucose was 47 mg/dL (reference range: 40–76 mg/dL), and the white cell count was found 1 cell/ mm^3^ (100% lymphocytes). Oligoclonal bands were not made. The brain MRI showed signal hyperintensity in T1 and FLAIR at the level of both mesial temporal lobes (Figure 1), hippocampi, and in the head of the right caudate nucleus (Figure 2). The thyroid stimulating hormone (TSH) level was 2.83 μIU/mL (reference range: 0.50–5.0 μI/mL) and thyroxine (T4) was 1.09 ng/dL (reference range: 0.93–1.71 μIU/mL). The serum anti-thyroglobulin (anti-Ty) level was 139.6 IU/mL (reference range: <20 IU/mL), and anti-thyroid peroxidase was 268.3 IU/mL (reference range: <35 IU/mL). Given these findings, HE was proposed as a diagnosis, therefore pulses of methylprednisolone were started. After that, there was partial improvement of epileptic seizures and involuntary movements. However, the hyponatremia could not be corrected. An antibody panel for autoimmune encephalitis was requested, supported by the neurological presentation of limbic encephalitis and the findings in the complementary exams.

**Figure 3:**
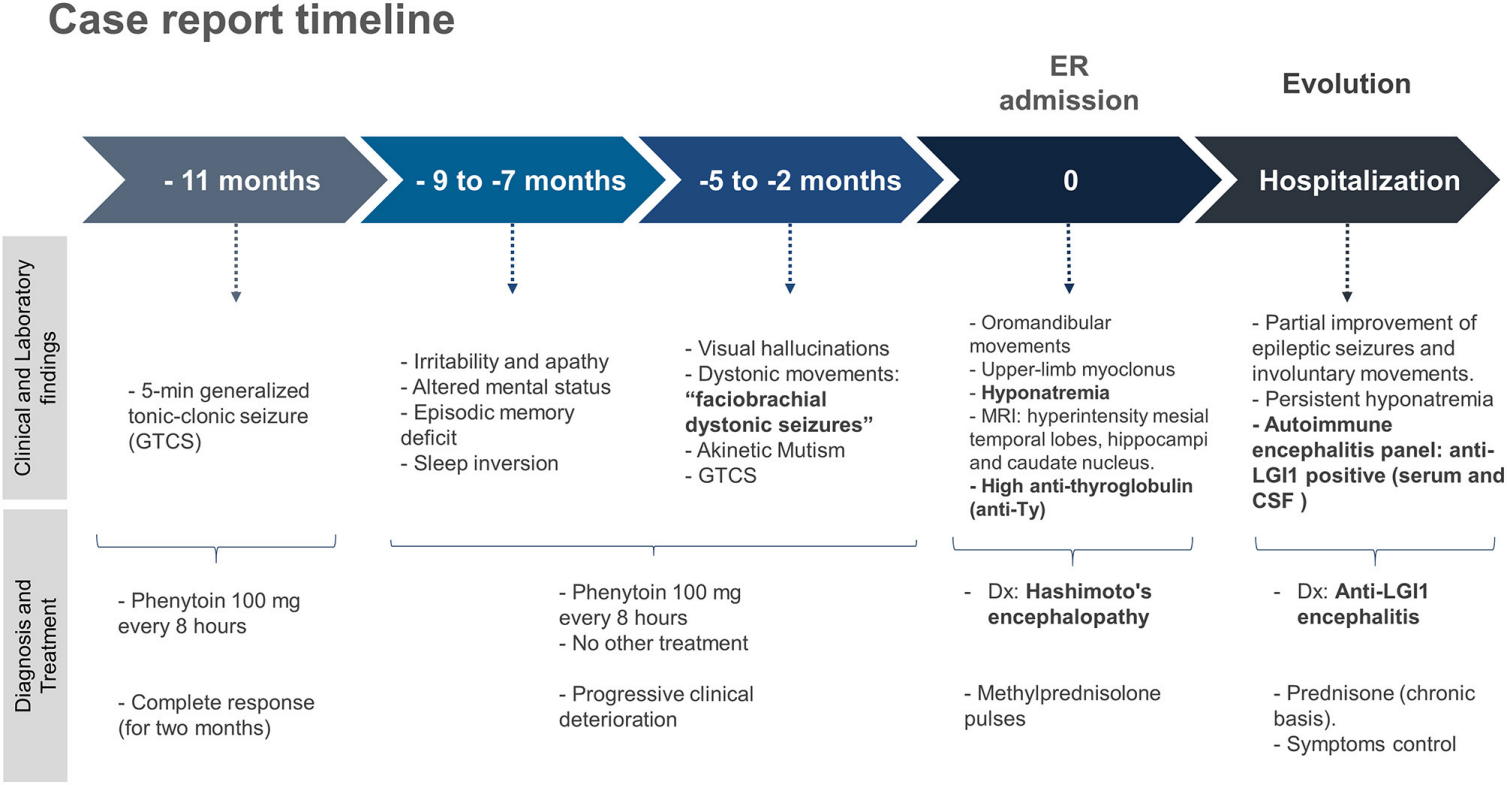
Case report timeline.

An antibody panel against autoimmune encephalitis was performed in serum and CSF, which included antibodies anti-N-methyl-D-aspartate receptor (NMDAr), anti-α-amino-3-hydroxy-5-methyl-4-isoxazolepropionic acid (AMPAR), acid receptor g-aminobutyric B (GABAB), and voltage-gated potassium channel (VGKC) complex components such as LGI1 and contactin-associated protein-like 2 (Caspr2). The anti-LGI1 antibody presence was positive in both serum and CSF. The patient received prednisone on a chronic basis, thus achieving better control of the symptoms. The case report timeline is presented in the Figure 3.

## Discussion

We present the case of a male patient with an onset of epileptic seizures associated with mental disorder, and affectation of higher functions with the presence of anti-Ty antibodies who was initially diagnosed as HE. However, the response to corticosteroids was not completed as it is usually observed in HE. This motivated the study of antibodies for autoimmune encephalitis, which was positive for anti-LGI1 antibodies.

Anti-LGI1 autoimmune encephalitis was first described in 2010. It occurs between the ages of 30 to 80 with a predominance in males between the sixth and seventh decade of life. It represents the most common cause of immune-caused limbic encephalitis as well as it is the second most frequent cause of autoimmune encephalitis after anti-NMDAr encephalitis (6). Because of our patient presented suggestive symptoms, a panel of antibodies was requested. However, the unavailability of the test in hospitals in our country and the high cost in private laboratories delayed its results. Despite the increasing number of cases of global anti-LGI1 encephalitis are being reported, our case is the first reported in our country. We believe that poor access to diagnostic testing underestimates the true frequency of autoimmune encephalitis in most Latin American countries.

Our patient also presented involuntary movements that fit the description of FBDS. The nature of these crises is debatable, since some authors consider it to be of subcortical origin while others defend its cortical origin in relation to epileptic seizures. They are described as contractions of one side of the face with flexion of the shoulder, arm, wrist, and ipsilateral finger extension that last <3 s and occur several times during the day (7). They usually precede cognitive decline and GTCS. However, in our case, the patient debuted with GTCS, and FBDS that occurred at 6 months of illness along with cognitive decline. This manifestation is almost pathognomonic of this diagnosis, it happens between 20 and 70% of cases (8, 9). Besides in a series of 13 cases by Wang et al. indicated that all of his patients presented this finding (6). Therefore, it is important to recognize this clinical manifestation and its relationship with anti-LGI1 encephalitis.

Other important findings described in anti-LGI1 encephalitis are hyponatremia, mental disorders, and sleep alterations (5). Hyponatremia is reported in up to 65% of cases and it is usually refractory to treatment (3), probably in association with a syndrome of inappropriate antidiuretic hormone secretion due to the presence of LG1 receptors in the hypothalamus and kidney. In our patient, sodium levels could not be normalized during their hospital stay. Although there was a slight increase in blood sodium level after the start of corticosteroid therapy. This is aligned with the literature showing that the blood sodium level reflects the severity of the disease (9); since our patient was severely compromised.

Behavioral disorders can be very diverse. Our patient presented apathy and irritability. Apathy is reported in up to 54%, while the frequency of organic irritability or excitation was presented in 39% of the cases (6). The EEG continuous generalized slowing of the background activity, without epileptogenic discharges, and the CSF study showed no abnormalities, which is consistent with previous the literature (4, 6).

The brain MRI presented hypersignals in both hippocampi bilaterally in FLAIR, in addition to hypersignal in T1 and FLAIR int right BG; these findings were described in previous case series (4, 6). It should be noted that in previous reports the unilateral hypersignal in BG (in FLAIR), correlates with contralateral FBDS. In our case, this hypersignal was ipsilateral and not contralateral to his FBDS. However, it does not rule out that FBDSs on the left side could have gone unnoticed, which is frequent in this condition (6, 10).

One of the differential diagnosis is HE (9), also known as steroid-sensitive encephalopathy associated with autoimmune thyroiditis, which is a clinically heterogeneous entity, traditionally defined as an encephalopathy associated with the presence of antithyroid antibodies. Our patient was initially diagnosed as HE due to neurological and psychiatric symptoms, and the presence of anti-Ty antibodies. However, these antibodies have shown to be not very specific to define this disease, and a factor of overdiagnosis; since, they have also been evidenced in other immune-mediated disorders, such as some autoimmune encephalitis, and even in up to 13% of the normal population (11).

Tüzün et al. (12), detected anti-Ty antibodies in eight of 24 patients with limbic encephalitis, and in this group of patients the antineuronal antibodies were the most frequent finding. The low specificity of antithyroid antibodies to define HE and their coexistence with other antibodies that define various autoimmune encephalitis suggests that these are only part of an epiphenomenon triggered by the antineuronal antibody and forces the need to refocus studies toward the systematic exclusion of antineuronal antibodies in patients with symptoms suggestive of immune-mediated encephalitis; even in those with antithyroid antibodies. It is important to note that antibody testing should not be a requirement to start the treatment if there is a high clinical suspicion. Opposite behavior can have a significant negative impact, since it is known that the prognosis, especially cognitive, in this entity is related to timely treatment. Likewise, it is important to highlight that high doses of IV steroids followed by prolonged oral steroids seem to be more effective in LGI1 antibody encephalitis, as occurred in our case (10).

## Conclusion

Anti-LGI1 encephalitis is a non-frequent and poorly understood entity that represents the second most frequent cause of autoimmune encephalitis. It is distinguished by the presence of limbic encephalitis, hyponatremia, and FBDS. HE is an important differential diagnosis to consider. Furthermore, the presence of anti-Ty antibodies should not be taken as the definitive diagnostic criteria, since these antibodies could be associated with other autoimmune encephalopathies, which include in addition to anti-LGI1, anti-NMDA and anti-Caspr2. In resource-limited countries with difficult access to the antibody test, it should be noted that it may be cheaper than requesting repeat MRIs. Even more so considering that anti-LGI1 encephalitis presents characteristic findings in MRI that should not generate uncertainty.

## Data Availability Statement

The original contributions presented in the study are included in the article/supplementary material, further inquiries can be directed to the corresponding author/s.

## Ethics Statement

The studies involving human participants were reviewed and approved by National Institute of Neurological Sciences. The patients/participants provided their written informed consent to participate in this study.

## Author Contributions

RO-S, AC, and WD contributed to the conception and data collection. KP-B and DS contributed to developing the idea of the study and revising the article critically for important intellectual content. All authors contributed to the article and approved the submitted version.

## Conflict of Interest

The authors declare that the research was conducted in the absence of any commercial or financial relationships that could be construed as a potential conflict of interest.
